# Impact of cigarette versus electronic cigarette aerosol conditioned media on aortic endothelial cells in a microfluidic cardiovascular model

**DOI:** 10.1038/s41598-021-83511-7

**Published:** 2021-02-26

**Authors:** Om Makwana, Gina A. Smith, Hannah E. Flockton, Gary P. Watters, Frazer Lowe, Damien Breheny

**Affiliations:** 1 Covance Laboratories Ltd, Genetic and Molecular Toxicology, Harrogate, UK; 2 BAT R&D Centre, Southampton, UK; 3 Department of Internal Medicine, Section On Molecular Medicine, Wake Forest School of Medicine, Winston-Salem, NC 27157 USA

**Keywords:** Biological techniques, Cell biology

## Abstract

Atherosclerosis is a complex process involving progressive pathological events, including monocyte adhesion to the luminal endothelial surface. We have developed a functional in vitro adhesion assay using BioFlux microfluidic technology to investigate THP-1 (human acute monocytic leukaemia cell) monocyte adhesion to human aortic endothelial cells (HAECs). The effect of whole smoke conditioned media (WSCM) generated from University of Kentucky reference cigarette 3R4F, electronic cigarette vapour conditioned media (eVCM) from an electronic nicotine delivery system (ENDS) product (Vype ePen) and nicotine on monocyte adhesion to HAECs was evaluated. Endothelial monolayers were grown in microfluidic channels and exposed to 0–1500 ng/mL nicotine or nicotine equivalence of WSCM or eVCM for 24 h. Activated THP-1 cells were perfused through the channels and a perfusion, adhesion period and wash cycle performed four times with increasing adhesion period lengths (10, 20, 30 and 40 min). THP-1 cell adhesion was quantified by counting adherent cells. WSCM induced dose-dependent increases in monocyte adhesion compared to vehicle control. No such increases were observed for eVCM or nicotine. Adhesion regulation was linked to increased ICAM-1 protein expression. Staining of ICAM-1 in HAECs and CD11b (MAC-1) in THP-1 cells demonstrated adhesion molecule co-localisation in BioFlux plates. The ICAM-1 adhesion response to WSCM was downregulated by transfecting HAECs with ICAM-1 siRNA. We conclude that the BioFlux system is able to model human monocyte adhesion to primary human endothelial cells in vitro and WSCM drives the greatest increase in monocyte adhesion via a mechanism involving endothelial ICAM-1 expression.

## Introduction

Cardiovascular disease (CVD) encompasses a number of defined diseases including coronary heart disease (CHD), angina, carotid artery disease (plaque in neck arteries that supply blood to the brain) and peripheral artery disease (PAD; plaque in arteries of the extremities, especially the legs). CHD is defined as a narrowing of blood vessels that supply blood to the heart, most frequently caused by the chronic and progressive build-up of plaque in the arteries. This build-up of arteriole plaque, also known as atherosclerosis, is a complex, multifaceted process involving several progressive pathological events leading to thrombosis^1^.

Several factors are known to increase the risk of developing cardiovascular disease including age, gender, high-fat diet, lack of exercise, hypertension, diabetes, and smoking^2^. Cigarette smoke yields many thousands of compounds, of which approximately 150 are known toxicants, and can cause many smoking-related diseases including cancer, respiratory and cardiovascular disease^3^. Cigarette smoke exposure causes chronic inflammation in the cardiovascular system and can lead to vascular endothelial cell dysfunction and endothelial cell death often associated with atherosclerosis^4–7^. Previous research has demonstrated cigarette smoke-induced cytotoxicity in a variety of human and mammalian endothelial cell types, including human umbilical vein endothelial cells (HUVECs) and primary human aortic endothelial cells (HAECs)^8^. Both apoptotic and necrotic mechanisms have been identified for smoking-induced endothelial cell death^7–12^.

A key early stage in the initiation of atherosclerosis involves circulating monocyte trafficking to the arterial endothelium in response to inflammation. The process of monocyte adhesion comprises upregulation of endothelial cell adhesion molecules such as E- and P-selectin, intercellular adhesion molecule-1 (ICAM-1), and vascular cell adhesion molecule-1 (VCAM-1) in lesion prone areas^13^. Selectins promote rolling along the endothelium and interact with P-selectin glycoprotein ligand-1 (PSGL-1), expressed at significantly higher levels by inflammatory monocytes. Endothelial VCAM-1 binds very late antigen-4 (VLA-4) to mediate slow rolling on inflamed endothelium, facilitating transition between rolling and firm adhesion. Firm monocyte adhesion to the endothelium is mediated by chemokine (C–C motif) ligand 2 (CCL-2) and IL-8 and endothelial ICAM-1. Monocyte arrest and subsequent extravasation is dependent on monocyte-expressed integrins lymphocyte function-associated antigen-1 (LFA-1) and macrophage-1-antigen (Mac-1/CD11b) alongside endothelial ligands ICAM-1 and ICAM-2^13,14^. Once attached, monocytes undergo extravasation, sub-endothelial accumulation and differentiation into macrophages. Over time, these cells give rise to lipid-laden pathogenic foam cells and initiate formation of atherosclerotic plaques^13^.

Cigarette smoke has been shown to induce monocyte activation, increase levels of circulating leukocytes and increase leukocyte recruitment and adhesion to the endothelium^15^. Endothelial cells exposed to aqueous cigarette smoke extract (CSE) express increased levels of pro-inflammatory adhesion molecules ICAM-1, E-selectin, VCAM-1 and CCL2^16^, causing increased monocytic MM6 cell adhesion to HUVECs^17^. Poussin et al. demonstrated a greater TNFα-induced monocyte adhesion response following HUVEC exposure to conditioned media from MM6 cells treated with CSE compared to direct HUVEC exposure to CSE^17^. Further work demonstrated higher concentrations of CSE are required to induce monocyte adhesion to human coronary artery endothelial cells (HCAECs) after direct endothelial cell treatment whereas lower concentrations are required following treatment with monocyte-conditioned media^18^. Kalra et al. demonstrated that adhesion of peripheral blood monocytes to bovine aortic endothelial cells (BAEC) or HUVECs increased following exposure to cigarette smoke condensate (CSC)^19^. A concomitant increase in CD11b expression on the monocyte surface was noted. Treatment with nicotine demonstrated no such effects^19^. HUVEC exposure to CSC increased endothelial ICAM-1 and E-selectin expression. Monocyte-to-endothelial cell adhesion was inhibited by treatment with monoclonal antibodies to CD11b, ICAM-1 and E-selectin^19^. Other work has linked cigarette smoke exposure-induced monocyte-to-endothelial cell adhesion to increased CD11b/ICAM-1 expression but not VCAM-1 or E-selectin expression^20,21^.

One strategy to reduce the harmful side effects of tobacco smoking is the development of next-generation tobacco and nicotine products (NGPs) such as electronic cigarettes (e-cigarettes) and tobacco heating products (THPs). Research by various groups has demonstrated that NGPs produce reduced levels of toxicants compared to cigarettes^22,23^. Recent comparisons between total particulate matter (TPM) generated from a 3R4F cigarette against TPM from a commercial THP, e-cigarette liquid (e-liquid) and aerosol produced by the same e-liquid found that THPs produce negative results in standard genotoxicity tests including the mouse lymphoma assay and in vitro micronucleus assay, whilst 3R4F TPM induced mutations in both assays^24,25^.

A recent review of the effects of e-cigarettes on cardiovascular health detailed several in vitro and in vivo studies which suggest that markers of oxidative stress, inflammation, vascular dysfunction and thrombosis were adversely affected by exposure to e-cigarettes, although to a lesser degree than tobacco smoke exposure^26,27^. A small study of 40 healthy subjects found smoking either a traditional cigarette or an e-cigarette was associated with increased oxidative stress markers and adversely effected brachial artery flow-mediated dilatation (an in vivo test of endothelial function), although e-cigarettes appeared to have lesser impact^28^. An in vitro study also found that incubation of platelets with either tobacco smoke or e-cigarette vapour extracts, led to upregulation of inflammatory markers and increased platelet activation, aggregation and adhesion^29^. However, the effect of NGPs on cardiovascular endothelial cell function, inflammation and monocyte activation still requires much greater investigation.

It has previously been suggested that the most appropriate cardiovascular model for cigarette smoke exposure would incorporate appropriate cardiovascular cell type(s), exposed to whole smoke bubbled media (containing both the particulate and vapour phase constituents) and would recapitulate in vivo vascular physiology by incorporating shear flow^30^. This model must demonstrate a correlation between clinical and in vitro effects of cigarette smoke exposure^30^ and as chronic human diseases (such as CVD) manifest over several years to decades, the practical limitations of exposure for any in vitro model must be considered. These same criteria can also be applied to examine exposure of the cardiovascular system to vapour from NGPs.

The BioFlux system is a high content screening platform for running directional flow assays at precisely controlled, automated shear rates combined with high resolution fluorescent microscopy using well plate microfluidic technology. It combines the biological relevance of a laminar flow cell set-up with the convenience and form factor of standard laboratory 24- or 48-well microplates, by embedding micron-scale fluidic channels on the plate bottom. The system is fully integrated and does not require manual control of shear pressure regulation or flow direction as in traditional microfluidic systems. An automated microscope stage allows controlled imaging at pre-defined channel locations supporting experimental reproducibility and consistency. By controlling the liquid flow rate through the fluidic channels (shear flow), the system can simulate a physiologically relevant environment for endothelial cells which depend upon shear flow to maintain homeostasis. Previous studies have examined vascular endothelial cell responses under conditions that partially recapitulate physiological context using BioFlux automated microfluidic technology^31–33^. In vitro CVD models have been used in both the absence and presence of shear flow^34^. Cockcroft and colleagues demonstrated an in vitro cardiovascular model that utilised HUVECs and a variety of flow patterns to model TNFα-induced inflammatory responses^35^. Similarly, others have used bespoke in vitro systems to deliver varying rates of shear flow to BAECs^36–38^, HUVECs^39^ and, more recently HAECs^40–42^. High shear flow has an atheroprotective effect on the endothelium and reduces endothelial cell response to cardiovascular risk factors compared to regions exposed to low or disturbed shear flow^43^. A previous study found that monocyte-to-endothelial cell adhesion is increased by CSE under low laminar flow but not under high laminar flow conditions^16^.

Here, we evaluate the effects of whole smoke conditioned media (WSCM) generated from University of Kentucky reference cigarette 3R4F, electronic cigarette vapour conditioned media (eVCM) from an electronic nicotine delivery system (ENDS) product (Vype ePen) and nicotine on human monocytic leukaemia (THP-1) cell adhesion to HAECs using the BioFlux microfluidic system. To investigate the mechanism of adhesion, cellular adhesion molecules (ICAM-1, MAC1/CD11b and E-selectin) were examined by immunofluorescence microscopy and western blotting, and subsequent modulation of the adhesion response was performed by transfecting HAECs with ICAM-1 siRNA prior to WSCM, eVCM or nicotine treatment.

## Results

**Figure 1:**
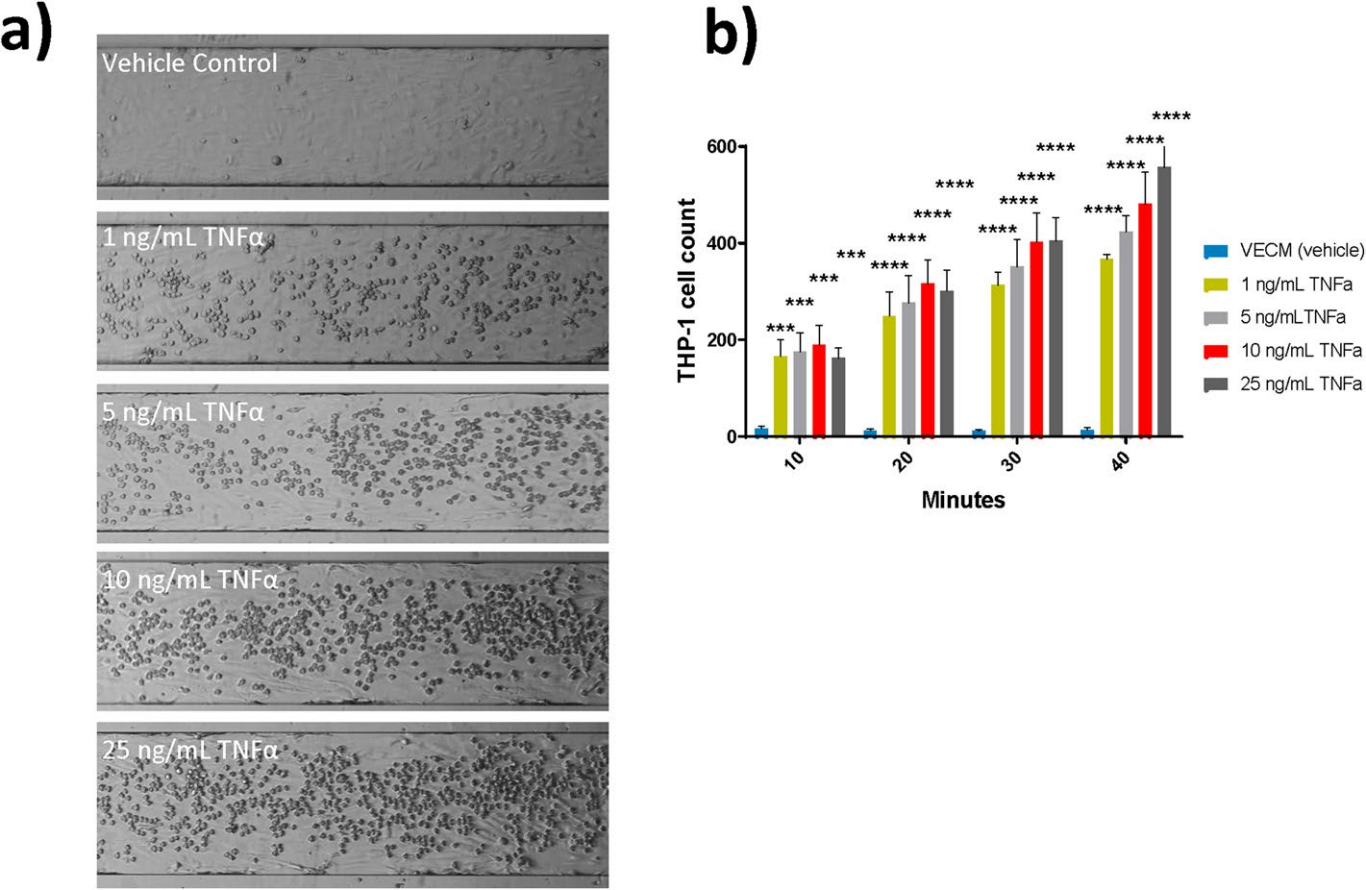
Monocyte adhesion to HAECs following 24 h TNFα exposure in the BioFlux microfluidic system. (**a**) Representative bright field images of BioFlux microfluidic channels captured after the 40 min adhesion period using a Zeiss Axio Observer.Z1 microscope (×10 magnification). (**b**) Quantification of monocyte adhesion to HAECs over a course of adhesion periods (10, 20, 30 and 40 min) following 24 h HAEC exposure to a TNFα dose range (1–25 ng/mL). Two-way ANOVA with Dunnett’s multiple comparisons between vehicle control and each TNFα concentration. Error bars denote ± SD (n = 3); ***p < 0.001 ****p < 0.0001.

To determine the feasibility of using the BioFlux microfluidic device as an appropriate tool for modelling cardiovascular disease-related events in vitro, HAEC monolayers were grown in BioFlux microfluidic channels and their response to pro-inflammatory cytokine, TNFα, assessed (Fig. 1). Images taken using phase contrast microscopy demonstrated that monocyte adhesion to HAECs was quantifiable in the BioFlux microfluidic system in response to TNFα treatment (Fig. 1a). An increase in monocyte adhesion to HAECs was observed with increasing TNFα concentration and adhesion period length in the BioFlux channels (Fig. 1b).

**Figure 2:**
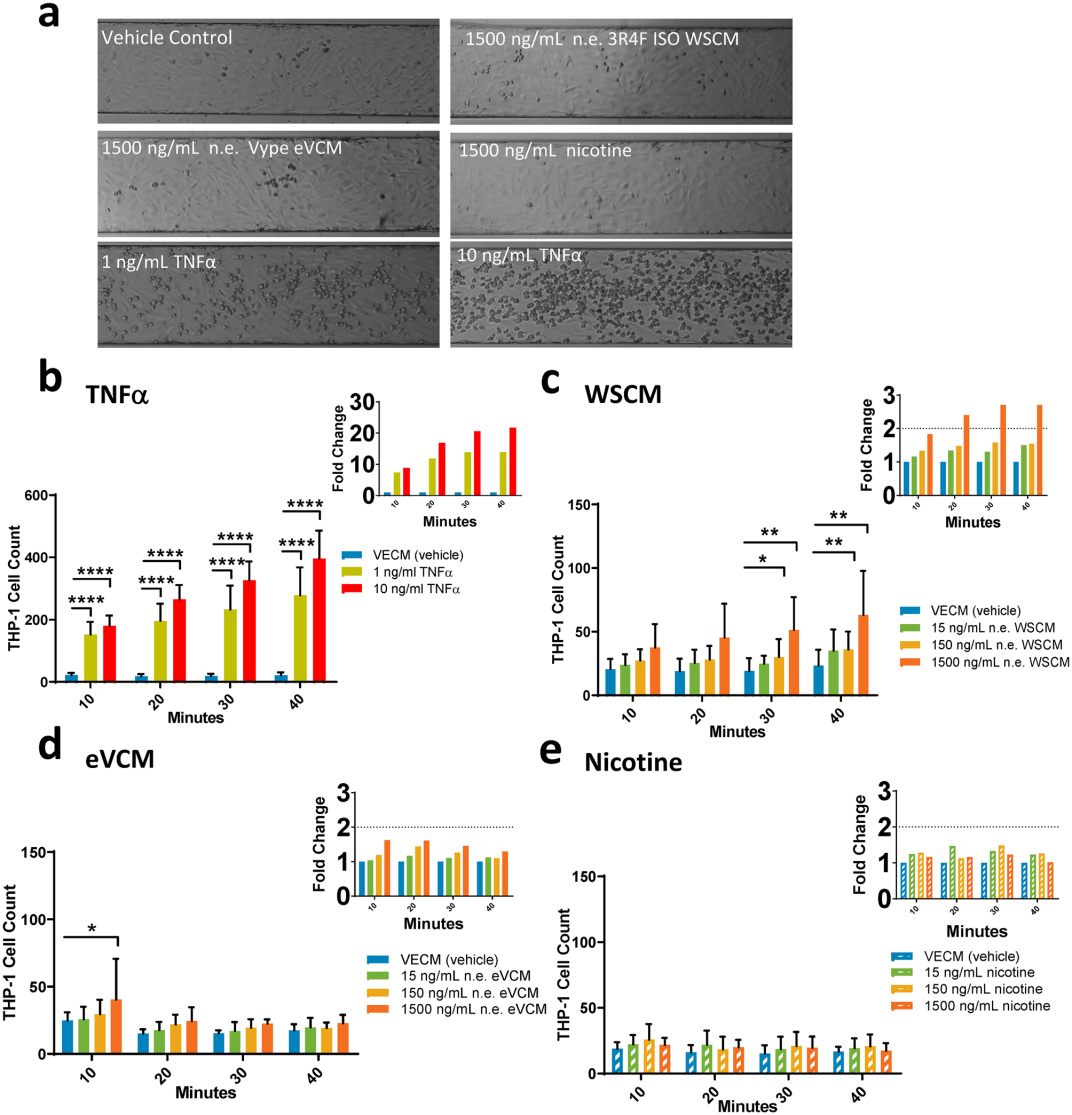
Monocyte adhesion to HAECs following 24 h test article exposure in the BioFlux microfluidic system. (**a**) Representative images of monocyte adhesion to HAECs following 24 h HAEC exposure to test article in BioFlux microfluidic channels. Brightfield images were captured after the 40 min adhesion period using a Zeiss Axio Observer.Z1 microscope (×10 magnification). Quantification of monocyte adhesion to HAECs over a course of adhesion periods (10, 20, 30 and 40 min) following 24 h HAEC exposure to a dose range of (**b**) TNFα, (**c**) WSCM, (**d**) eVCM or (**e**) nicotine in BioFlux microfluidic channels with fold change graphs inset. Two-way ANOVA with Dunnett’s multiple comparisons between vehicle control and each test article concentration. Error bars denote ± SD (n = 6), TNFα (n = 30); *p < 0.05 **p < 0.01 p < 0.001 p < 0.0001.

The impact of endothelial cell exposure to WSCM, eVCM or nicotine on THP-1 cell adhesion was evaluated across a range of non-toxic concentrations (15, 150 and 1500 ng/mL n.e.^8^) for 24 h alongside concurrent vehicle control, and 1 and 10 ng/mL TNFα as positive adhesion controls (Fig. 2). Representative channel images demonstrate THP-1 cell adhesion to HAECs after the final (40 min) adhesion period and wash step (Fig. 2a). THP-1 cell adhesion increased from 7.4-fold at the 10-min adhesion period to 13.8-fold at the 40-min adhesion period in response to HAEC treatment with 1 ng/mL TNFα compared to the concurrent vehicle control (Fig. 2b). THP-1 cell adhesion increased from 8.9-fold at the 10-min adhesion period to 21.7-fold at the 40-min adhesion period in response to HAEC treatment with 10 ng/mL TNFα compared to the concurrent vehicle control (Fig. 2b). WSCM-treated HAECs demonstrated a concentration-related increase in THP-1 cell adhesion after each incubation period compared to concurrent vehicle control-treated HAECs. WSCM (1500 ng/mL n.e.) induced an approximate threefold increase in THP-1 cell adhesion to HAECs after the 40-min incubation period, compared to the concurrent vehicle control (Fig. 2c). HAEC treatment with 15 or 150 ng/mL n.e. WSCM induced an approximate 1.5-fold increase in THP-1 cell adhesion after the 40-min incubation period, compared to the concurrent vehicle control (Fig. 2c).

HAEC exposure to eVCM resulted in a 1.6-fold increase in THP-1 cell adhesion at the highest exposure concentration (1500 ng/mL n.e.) after the 10-min adhesion period (Fig. 2d) however, after the 20, 30 and 40-min adhesion periods, there was no significant (p > 0.05) increase in THP-1 cell adhesion observed across all of the exposure concentrations (Fig. 2d). Nicotine demonstrated no detectable effect on THP-1 cell adhesion to HAECs at equivalent concentrations and adhesion periods (Fig. 2e).

**Figure 3:**
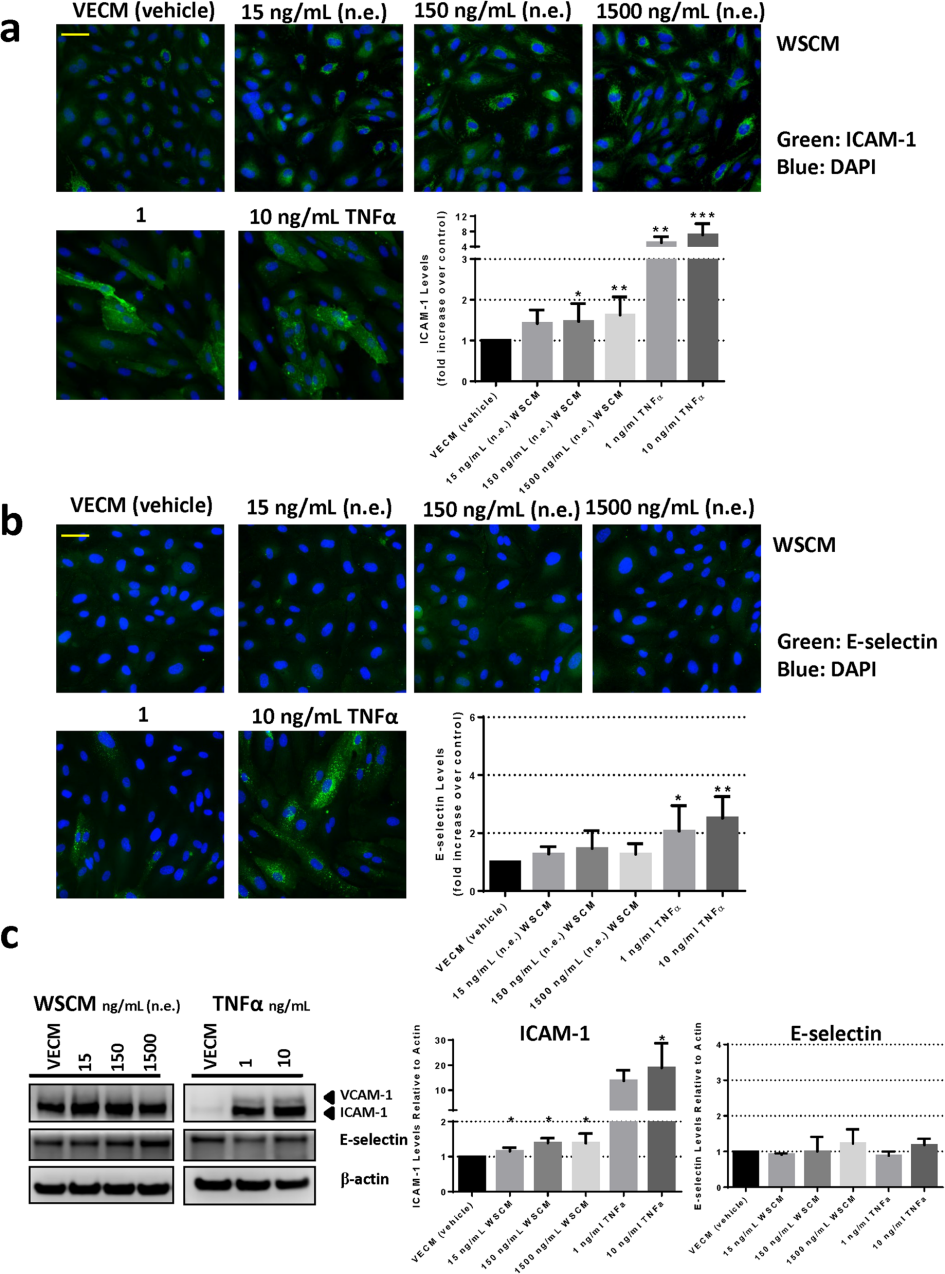
Analysis of ICAM-1 and E-selectin expression in HAECs following 24 h exposure to WSCM. (**a**) Immunofluorescence microscopy analysis and associated quantification of ICAM-1 or (**b**) E-selectin expression following HAEC exposure to WSCM for 24 h in Ibidi chamber slides. Images were captured using a Zeiss Axio Observer.Z1 microscope (×20 magnification). Scale bar (yellow line) = 70 µm. Errors bars denote ± SD (n = 6). (**c**) Western blot analysis and associated quantification of ICAM-1, VCAM-1 and E-selectin expression in HAECs following 24 h exposure to WSCM (refer to Supplementary Figure S1 for uncropped blots). One-way ANOVA with Dunnett’s multiple comparisons between vehicle control and each WSCM concentration. Error bars denote ± SD (n = 3); *p < 0.0 **p < 0.01 ***p < 0.001.

**Figure 4:**
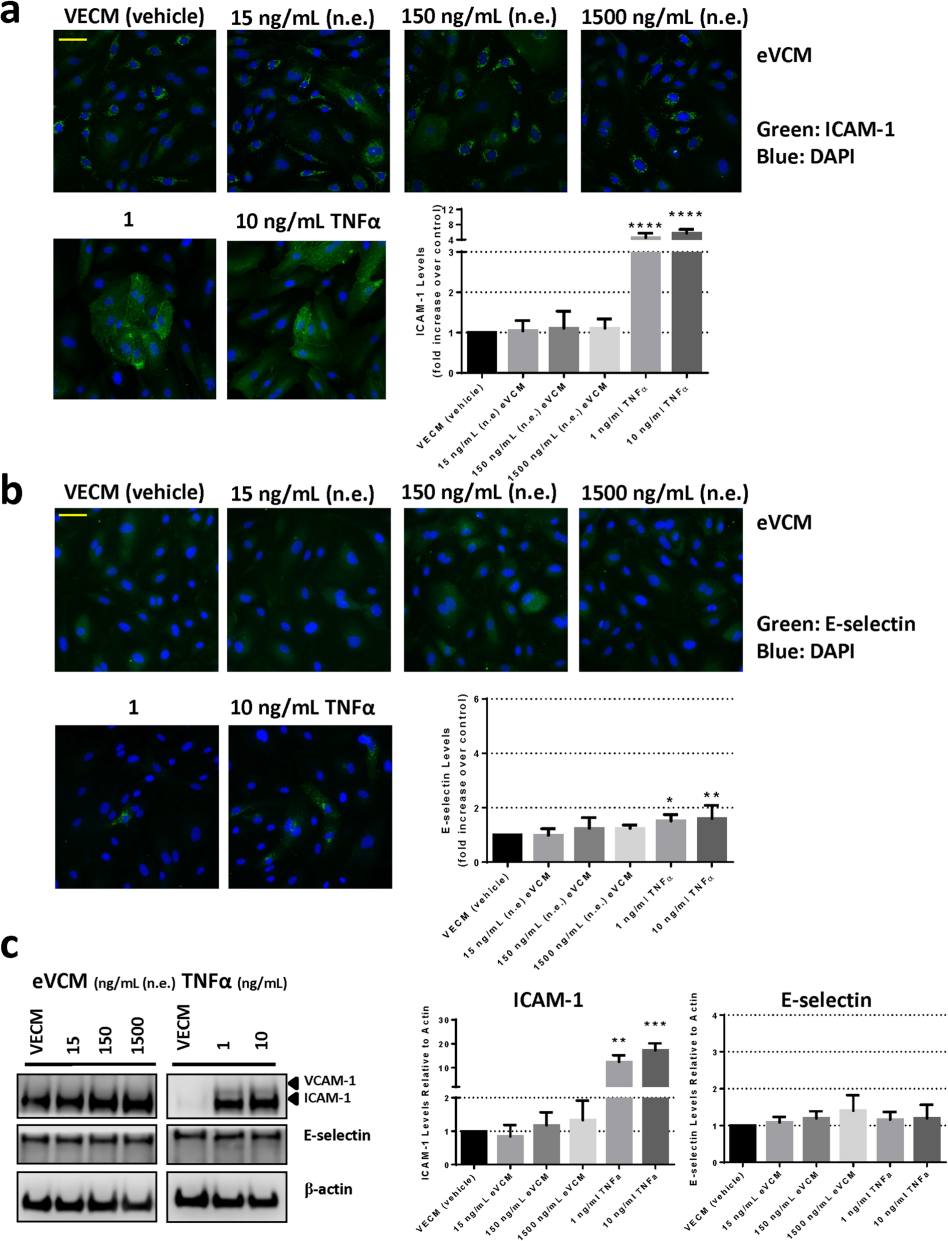
Analysis of ICAM-1 and E-selectin expression in HAECs following 24 h exposure to eVCM. (**a**) Immunofluorescence microscopy analysis and associated quantification of ICAM-1 or (**b**) E-selectin expression following HAEC exposure to eVCM for 24 h in Ibidi chamber slides. Images were captured using a Zeiss Axio Observer.Z1 microscope (×20 magnification). Scale bar (yellow line) = 70 µm. Errors bars denote ± SD (n = 6). (**c**) Western blot analysis and associated quantification of ICAM-1, VCAM-1 and E-selectin expression in HAECs following 24 h exposure to eVCM (refer to Supplementary Figure S1 for uncropped blots). One-way ANOVA with Dunnett’s multiple comparisons between vehicle control and each eVCM concentration. Error bars denote ± SD (n = 3); *p < 0.05 **p < 0.01 ***p < 0.001 ****p < 0.0001.

**Figure 5:**
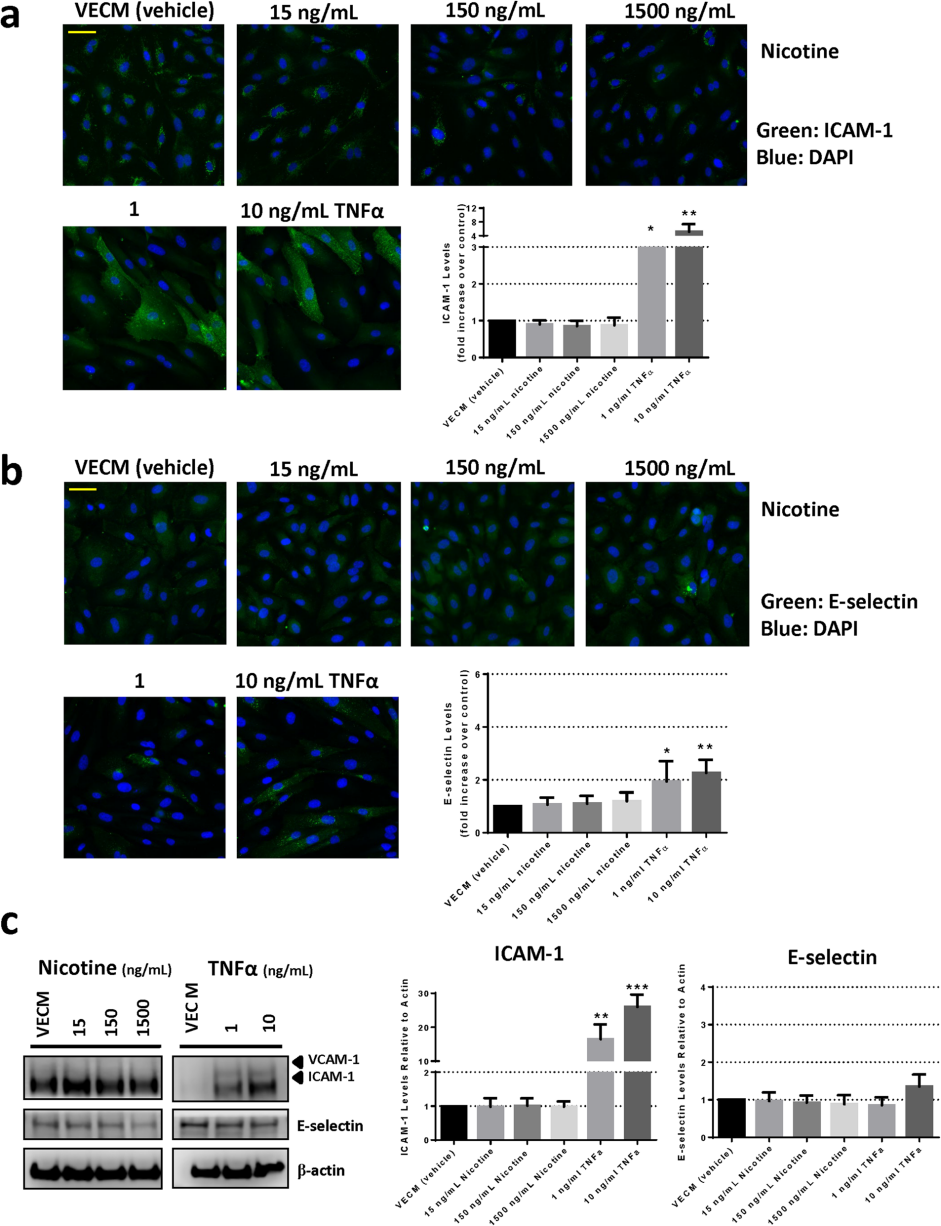
Analysis of ICAM-1 and E-selectin expression in HAECs following 24 h exposure to nicotine. (**a**) Immunofluorescence microscopy analysis and associated quantification of ICAM-1 or (**b**) E-selectin expression following HAEC exposure to nicotine for 24 h in Ibidi chamber slides. Images were captured using a Zeiss Axio Observer.Z1 microscope (×20 magnification). Scale bar (yellow line) = 70 µm. Errors bars denote ± SD (n = 6). (**c**) Western blot analysis and associated quantification of ICAM-1, VCAM-1 and E-selectin expression in HAECs following 24 h exposure to nicotine (refer to Supplementary Figure S1 for uncropped blots). One-way ANOVA with Dunnett’s multiple comparisons between vehicle control and each nicotine concentration. Error bars denote ± SD (n = 3); *p < 0.05 **p < 0.01 ***p < 0.001.

The mechanism of monocyte adhesion to HAECs was first investigated using immunofluorescence microscopy and Western blotting for ICAM-1, VCAM-1 and E-Selectin protein expression in HAECs after WSCM, eVCM or nicotine exposure (Fig. 3, 4, 5). Using immunofluorescence microscopy methodology, mean ICAM-1 expression increased 4.4-fold and sixfold in response to HAEC treatment with 1 ng/mL and 10 ng/mL TNFα, respectively in comparison to vehicle control (Figs. 3a, 4a, 5a). Mean E-selectin expression increased 1.8-fold and 2.1-fold in response to HAEC treatment with 1 ng/mL and 10 ng/mL TNFα, respectively in comparison to vehicle control (Figs. 3b, 4b, 5b). A concentration-related increase in endothelial ICAM-1 levels was observed in response to WSCM exposure for 24 h. HAEC treatment with 150 and 1500 ng/mL n.e. WSCM induced significant (p < 0.05) increases in ICAM-1 expression of approximately 1.5-fold and 1.6-fold, respectively (Fig. 3a). No such increases in E-selectin or VCAM-1 levels were observed (Fig. 3a–c). There was no significant increase in ICAM-1 or E-selectin expression in HAECs following eVCM (Fig. 4a,b) or nicotine exposure (Fig. 5a,b).

These data were confirmed via Western blot methodology, where a concentration-related increase in endothelial ICAM-1 levels was observed in response to WSCM exposure for 24 h. HAEC treatment with 150 or 1500 ng/mL n.e. WSCM induced an approximate 1.4-fold increase in ICAM-1 expression (Fig. 3c). No such increases in E-selectin or VCAM-1 levels were observed (Fig. 3a–c). There was no significant increase in ICAM-1 or E-selectin expression in HAECs following eVCM (Fig. 4c) or nicotine exposure (Fig. 5c). In comparison, mean ICAM-1 expression increased 14.2-fold and 20.7-fold in response to HAEC treatment with 1 ng/mL and 10 ng/mL TNFα, respectively compared to vehicle control. Mean E-selectin expression increased 1.4-fold in response to HAEC treatment with 10 ng/mL TNFα (Figs. 3c, 4c, 5c). Quantifiable levels of VCAM-1 were observed in TNFα-treated HAECs only (Figs. 3c, 4c, 5c).

**Figure 6:**
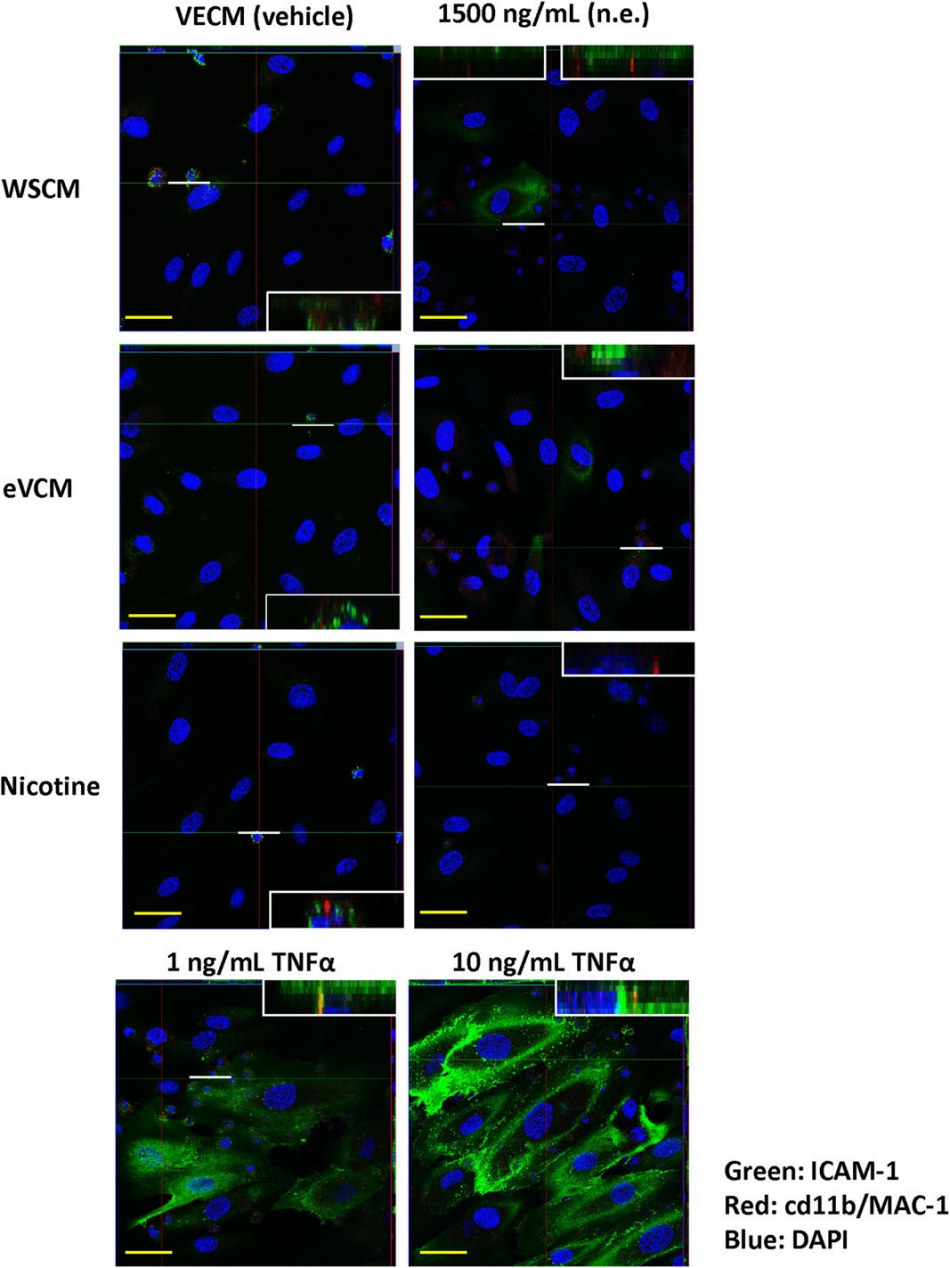
Analysis of ICAM-1 and CD11b co-localisation in HAECs following 24 h exposure to test article in the BioFlux microfluidic system. Immunofluorescence microscopy analysis of ICAM-1 and CD11b/MAC-1 co-localisation between monocytes and HAECs following 24 h WSCM, eVCM or nicotine exposure in BioFlux microfluidic channels. Monocyte adhesion to HAECs was assessed over a course of adhesion periods (10, 20, 30 and 40 min). Images were captured after the 40 min adhesion period using a Zeiss LSM780 confocal microscope (×40 magnification). Scale bar (yellow line) = 35 µm. To assess co-localisation, z-planes were applied through the composite z-stack. Inset images represent z-planes from regions marked with a white line.

The ICAM-1-mediated THP-1 cell-to-HAEC adhesion response was reproduced in BioFlux microfluidic channels treated with 0 or 1500 ng/mL WSCM, eVCM or nicotine for 24 h and processed for confocal microscopy using antibodies to endothelial ICAM-1 and monocyte-expressed CD11b. This demonstrated upregulation of ICAM-1 and evidence of interaction with adhered THP-1 cells via CD11b after HAEC exposure to TNFα or WSCM but not after exposure to eVCM or nicotine (Fig. 6).

**Figure 7:**
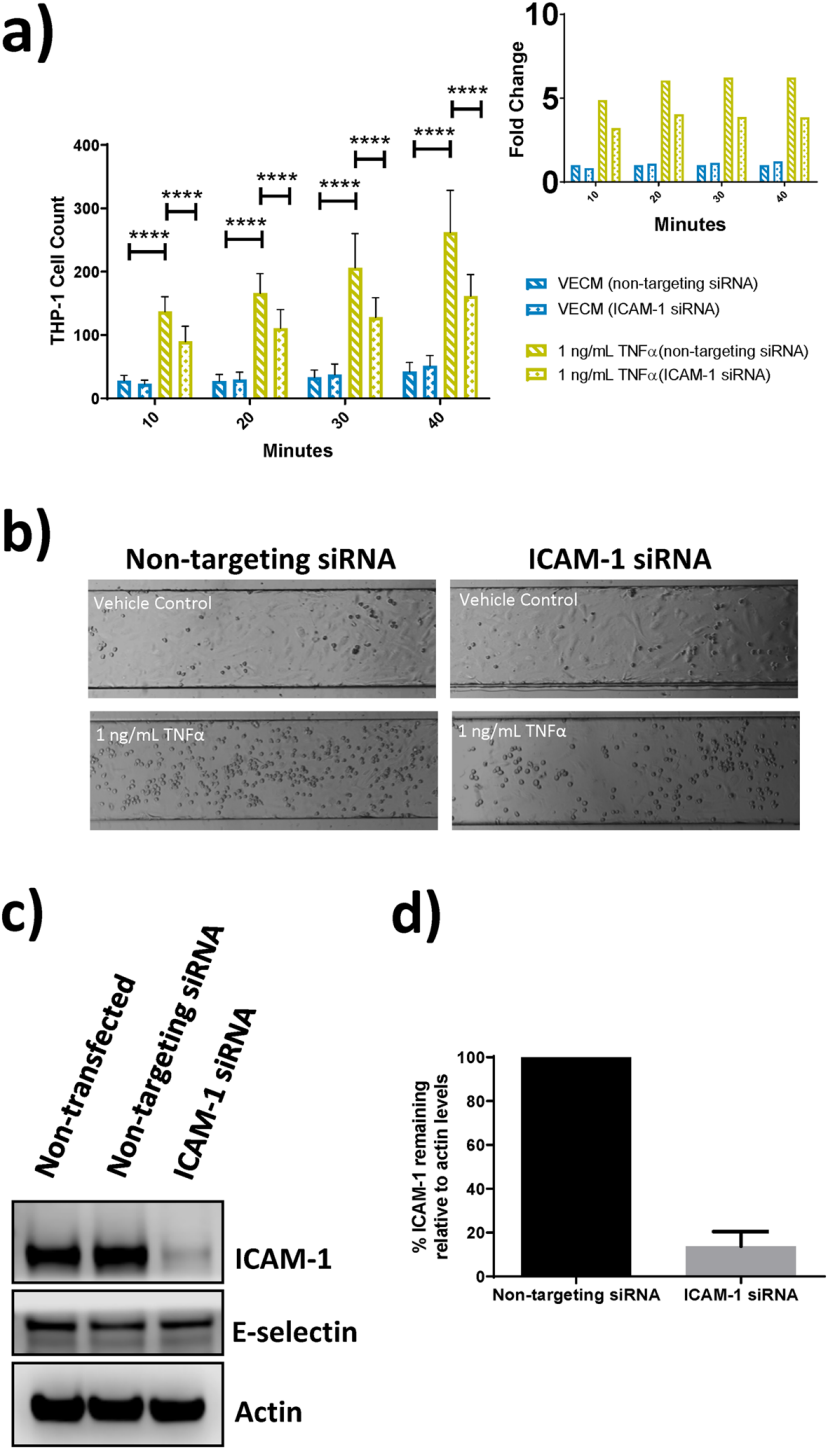
Analysis of ICAM-1 involvement in TNFα-induced monocyte adhesion to HAECs in the BioFlux microfluidic system. (**a**) Quantification of monocyte adhesion to HAECs over a course of adhesion periods (10, 20, 30 and 40 min) following transfection with control or ICAM-1 siRNA and 24 h HAEC exposure to 1 ng/mL TNFα in BioFlux microfluidic channels, with fold change graphs inset. (**b**) Representative brightfield images of BioFlux microfluidic channels captured after the 40 min adhesion period using a Zeiss Axio Observer.Z1 microscope (×10 magnification) following transfection with control or ICAM-1 siRNA and 24 h HAEC exposure to TNFα. (**c**) Western blot analysis of ICAM-1 and E-selectin levels following 72 h transfection of HAECs with control or ICAM-1 siRNA (refer to Supplementary Figure S1 for uncropped blots). (**d**) Quantification of ICAM-1 levels following 72 h transfection of HAECs with control or ICAM-1 siRNA. Two-way ANOVA with Dunnett’s multiple comparisons between vehicle all treatment conditions. Error bars denote ± SD (n = 22); ****p < 0.0001.

ICAM-1 siRNA was utilised to deplete endothelial ICAM-1 levels. THP-1 cell adhesion to ICAM-1-depleted HAECs was significantly (p < 0.001) reduced in response to 1 ng/mL TNFα (Fig. 7a,b). Treatment of non-targeting siRNA-transfected HAECs with 1 ng/mL TNFα induced a sevenfold increase in THP-1 cell adhesion after the 40-min adhesion period in comparison to vehicle control (Fig. 7a). THP-1 cell adhesion to ICAM-1-depleted HAECs treated with 1 ng/mL TNFα was reduced by 38.3% compared to equivalent non-targeting siRNA-transfected HAECs (Fig. 7a,b). ICAM-1 knockdown was confirmed by Western blot (Fig. 7c) with quantification revealing an 86.2% knockdown efficiency (Fig. 7d). These results confirmed the role of ICAM-1 in TNFα-induced monocyte adhesion to HAECs.

**Figure 8:**
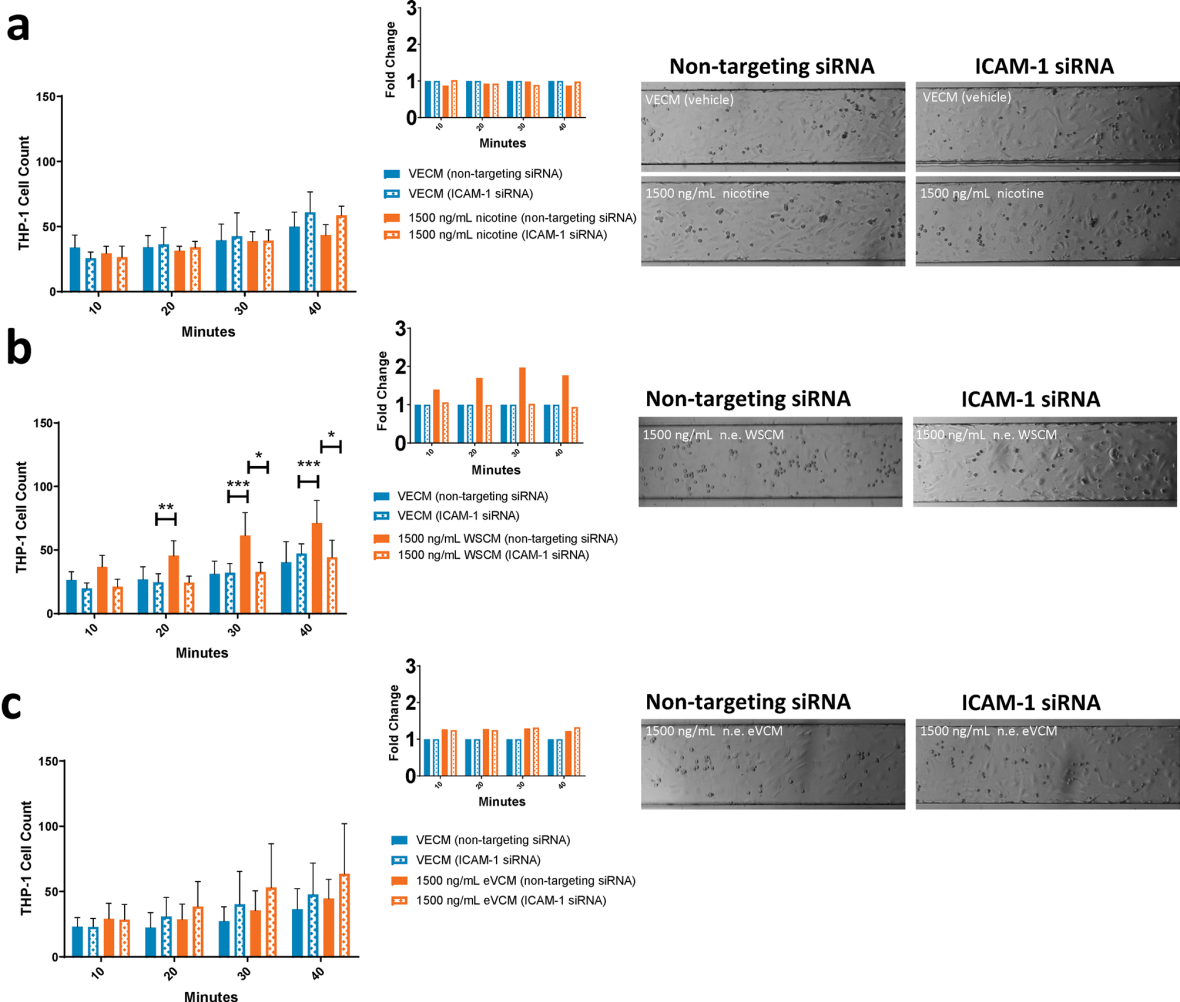
Analysis of ICAM-1 involvement in test article-induced monocyte adhesion to HAECs in the BioFlux microfluidic system. Quantification of monocyte adhesion to HAECs over adhesion periods of 10, 20, 30 and 40 min following 72 h transfection with control or ICAM-1 siRNA and 24 h HAEC exposure to (**a**) nicotine, (**b**) WSCM or (**c**) eVCM, with fold change graphs inset and representative brightfield images of BioFlux microfluidic channels captured after the 40 min adhesion period using a Zeiss Axio Observer.Z1 microscope (×10 magnification). Two-way ANOVA with Dunnett’s multiple comparisons between all treatment conditions. Error bars denote ± SD (n = 6); *p < 0.05 **p < 0.01 ***p < 0.001.

To confirm that WSCM-induced THP-1 cell adhesion to HAECs occurs via a similar ICAM-1-dependent mechanism, ICAM-1 siRNA transfected HAECs were treated with 1500 ng/mL n.e. WSCM, 1500 ng/mL n.e. eVCM or 1500 ng/mL nicotine (Fig. 8). WSCM treatment of HAECs transfected with non-targeting siRNA induced an approximate twofold increase in THP-1 cell adhesion versus the concurrent vehicle control after the 40-min incubation period, however this WSCM-related increase did not occur in HAECs transfected with ICAM-1 siRNA and THP-1 cell adhesion levels were comparable with vehicle control-treated HAECs (Fig. 8b). Thus, WSCM-induced monocyte adhesion to HAECs in BioFlux microfluidic channels appears to be driven by ICAM-1 (Fig. 8b). ICAM-1 depletion did not impact THP-1 cell adhesion to HAECs treated with eVCM or nicotine (Fig. 8a,c).

## Discussion

Cigarette smoke has been demonstrated to cause endothelial cell dysfunction^4^ and upregulation of monocyte adhesion, a key initiating step in the development of atherosclerosis^15–19^. Although NGPs are believed to be a less harmful alternative to smoking traditional cigarettes^23^, there is still more research required to understand the potential consequences of NGP use, particularly on cardiovascular health.

Here, we have demonstrated that THP-1 monocyte adhesion to HAECs in BioFlux microfluidic channels was statistically significantly increased after HAEC exposure to WSCM. Increased THP-1 cell adhesion to HAECs was noted across multiple WSCM concentrations (15, 150 and 1500 ng/mL n.e.), with increases up to approximately threefold in response to 1500 ng/mL (n.e.) WSCM. We have previously demonstrated that this concentration of WSCM exposure has no effect on HAEC viability^8^. In comparison, a much lower level of THP-1 cell adhesion to HAECs was observed after exposure to eVCM at equivalent nicotine concentrations (up to 1.6-fold), with minimal response detected up to the final 40-min adhesion period. The observed effects were based on multiple independent WSCM and eVCM batches generated in compliance with Good Laboratory Practice. HAEC exposure to nicotine induced no notable increase in THP-1 adhesion.

Further experimentation revealed that WSCM upregulates ICAM-1 levels in HAECs but not E-selectin or VCAM-1, measured by immunofluorescence microscopy and Western blot. No significant upregulation of ICAM-1, E-Selectin or VCAM-1 was detected following HAEC exposure to eVCM or nicotine. It should be noted that upon TNFα treatment, E-selectin re-distributed into intense fluorescent regions on the immunofluorescence microscopy images. This resulted in a higher fluorescence reading (arbitrary fluorescence units) compared to vehicle and WSCM treatments where E-Selectin staining was more diffuse. Western blot data indicated there was no change in E-selectin protein levels following 24 h TNFα treatment, thus cellular redistribution was considered to account for differences in the results between the two detection methodologies. Confocal microscopy analysis detected co-localisation between ICAM-1 and monocyte-expressed CD11b. This co-localisation occurred in regions of monocyte-to-endothelial cell adhesion, predominantly involving HAECs with upregulated ICAM-1 expression in response to WSCM or TNFα. The CD11b integrin is known to bind ICAM-1 in response to inflammation^13^. Transfection of HAECs with ICAM-1 siRNA provided further evidence for the role of ICAM-1 in WSCM-induced monocyte adhesion to HAECs. ICAM-1 depletion reduced THP-1 cell adhesion in response to WSCM to levels similar to those observed in the vehicle control. ICAM-1 depletion did not result in any such reductions in THP-1 cell adhesion to HAECs treated with eVCM or nicotine. Thus, WSCM-induced THP-1 cell adhesion to HAECs was preferentially ICAM-1-mediated.

Although no controlled shear pressure was applied during the monocyte adhesion periods, cells in the microfluidic channels were subject to gravity flow at all times. In an attempt to model a chronic condition that would otherwise occur over many years in a physiological setting and to model an inflammatory background in which quantifiable monocyte adhesion responses could be observed, we opted to use low shear rates to test the effects of smoke-conditioned media^43^. Previous work has demonstrated that high shear rates can be used to model a ‘healthy’ vascular environment in the BioFlux system^33^. Briefly, the effects of applied shear flow on primary HAECs in BioFlux microfluidic channels were investigated. Changes in cell and actin alignment in the direction of flow, real-time production of NO and gross cell membrane shape changes in response to physiological shear flow were observed^33^. These commercial systems have a range of potential applications, including within the consumer and pharmaceutical industries, thereby reducing the dependency on animal testing for regulatory safety assessments.

While in vitro CVD models have been used in both the absence and presence of shear flow, it is generally accepted that an ideal, dynamic in vitro CVD model would recapitulate in vivo vascular physiology by incorporating three dimensional (3D) shear flow. A wide range of in vitro microfluidic technologies encompassing 3D cell culture under flow have emerged with varying physiological relevance and complexity ^30,33,44–46^. These models can be used to study different aspects of vascular biology and offer varied advantages including: high throughput capacity, ease of setup and creation of a more physiological in vitro environment. They also come with limitations such as complex set-up, low throughput or lower physiological relevance^45,46^. It is important that the vascular model selected to define the biological mechanism of interest is considered for scalability, reproducibility, development potential and feasibility of use in addition to physiological relevance and throughput capacity. For example, the use of venous endothelial cells is less physiologically relevant then aortic cells to the study of atherosclerosis which predominantly occurs in the arteries however venous cells tend to be more widely available and have therefore been more commonly used in such in vitro models. Many labs assess monocyte adhesion in standard cell culture plate format or in plates with microfluidic channels that require a rocking platform to generate flow^45,46^. Our method utilises the commercially available BioFlux system and therefore offers a robust platform with high throughput capacity and reduced inherent variability to assess monocyte-to-endothelial cell adhesion at pre-defined, automated shear flows. Furthermore, in an attempt to more closely model atherosclerosis as a chronic and progressive condition the assay encompasses multiple adhesion periods of increasing length.

We conclude that the BioFlux microfluidic system may be a useful tool for modelling CVD in vitro to assess effects of both cigarette smoke and NGP vapour exposure. Our model encapsulates several of the criteria outlined by Fearon et al. for an appropriate cardiovascular model including a human-derived cardiovascular cell type (HAEC) and exposure to whole smoke or vapour bubbled media (WSCM and eVCM)^30^. The current assay design is relevant to a pathological environment in which shear flow is disturbed (close to zero)^47^. We have previously demonstrated that physiological shear flow induces cellular changes in HAECs in the BioFlux system^33^. To develop the monocyte-to-HAEC adhesion model further and to more accurately reproduce in vivo physiology, incorporation of constant shear flow could be explored. In conclusion, the THP-1 cell adhesion assay can be used to investigate the tobacco risk continuum^48^, as we observe consistently differential responses in monocyte-to-HAEC adhesion after exposure to WSCM, eVCM and nicotine.

## Methods

### Cell culture

Primary Human Aortic Endothelial cells (HAECs), were sourced from Lifeline Cell Technology (Frederick, MD, USA; Catalogue Number FC-0014, Lot Number 00503), and maintained in VascuLife VEGF Endothelial Cell Culture Medium supplemented with VEGF LifeFactors (VECM; Lifeline Cell Technology)^8,33^. Cell stocks were preserved in the vapour phase of liquid nitrogen, initiated from frozen, and maintained in culture until near confluence (approximately 70–90%)^8,33^. Cells were cultured at 37 ± 1 °C, in a humidified atmosphere of 5% (v/v) CO_2_ in air, and re-fed with VECM as required. HAECs at passage 5 were used in all experiments^8,33^.

Monocytic THP-1 cells, obtained from ATCC (Virginia, USA; Catalogue Number TIB-202), were maintained in RPMI 1640 media (Gibco, Billings, MT, USA) supplemented with 10% v/v heat-inactivated fetal bovine serum, penicillin and streptomycin. Cell stocks were preserved in the vapour phase of liquid nitrogen, initiated from frozen and maintained in culture with bi-weekly passage up to passage 30. Cells were cultured at 37 ± 1 °C, in a humidified atmosphere of 5% (v/v) CO_2_ in air, and re-fed with supplemented RPMI 1640 as required.

### WSCM or eVCM generation

3R4F Kentucky Reference (3R4F) cigarettes were obtained from the University of Kentucky and stored at < − 10 °C. Prior to smoking, cigarettes were removed from frozen storage and conditioned as individual cigarettes for at least 48 h and no more than 10 days at 22 ± 1 °C and 60 ± 3% relative humidity (ISO 3402, 1999)^8^. 3R4F cigarettes were smoked using a Borgwaldt RM200 smoking machine according to the puffing parameters of ISO 3308, 2012, namely a 35 mL puff every 60 s with a 2 s duration^8^.

Vype ePEN II and Vype blended tobacco ePen cartridges (18 mg/mL nicotine) (British American Tobacco) were stored at 25 ± 5 °C. Prior to puffing, e-cigarettes were held in the laboratory in airtight plastic containers, with caps in place, until exposure commenced. Vype ePEN II e-cigarettes were puffed using a VC10 smoking robot according to the puffing parameters of CORESTA CRM 81, namely a 55 mL square wave puff every 30 s with a 3 s duration.

For each WSCM or eVCM generation, whole smoke from four 3R4F cigarettes or e-vapour from one cartridge was passed through a 30 mL glass impinger containing approximately 6 g of 3 mm glass beads and 20 mL VECM^8^. WSCM or eVCM was filter sterilised under aseptic conditions using a 1000 mL Stericup-VP polyethersulfone Express PLUS, radio-sterilised 0.10 µm filter (Millipore, Sheffield, UK)^8^. WSCM or eVCM was stored frozen in 1 mL aliquots at < − 50 °C prior to use, analysed for nicotine content by gas chromatography with flame ionisation detector (GC-FID) and used within 4 weeks of generation^8^.

### BioFlux system

The BioFlux system (Fluxion; Alameda, CA, USA) is comprised of an air compressor and electropneumatic regulators to deliver precisely controlled pressure to a BioFlux plate via a pressure interface^33^. BioFlux plates are in a standard 24- or 48-well plate format with integrated microfluidic channels^33^. The bottom of each microfluidic channel consists of standard 180-μm coverslip glass, which allows microscopic examination at defined viewing windows^33^. Flow channels are connected to input and output wells from which reagents are pushed by pneumatic pressure through the channels^33^.

### BioFlux plate preparation and seeding

BioFlux microfluidic channels were coated with 100 µg/mL fibronectin, seeded with HAECs in VECM and incubated overnight under gravity flow conditions at 37 ± 1 °C in a humidified atmosphere of 5% CO_2_ in air, as described in Makwana et al.^33^.

### BioFlux HAEC treatment

Prior to treatment, brightfield images were taken at 3 fields of view per channel for reference monolayer confluence and morphology. VECM was removed from inlet and outlet wells and appropriate treatments vehicle control (VECM), positive control (1 and 10 ng/mL TNFα, Life Technologies, Altrincham, UK) or eVCM, WSCM or nicotine (Sigma-Aldrich) [0, 15, 150, 1500 ng/mL nicotine equivalent (n.e.)] were added to the inlet wells. HAECs were treated for 24 h in total. Treatments were perfused through the channels at 2 dyn/cm^2^ for 5 min to ensure appropriate distribution in the channel, then the plates were incubated under gravity flow conditions at 37 ± 1 °C in a humidified atmosphere of 5% CO_2_ in air for the remainder of the 24 h. Six experiments with separate generations of WSCM, eVCM or fresh preparations of nicotine were performed.

### THP-1 activation

THP-1 cells were counted and diluted in fresh culture medium to 1–2 × 10^5^ cells/mL in low adhesion cell culture flasks. The THP-1 cells were activated by addition of 0.1 ng/mL 12-O-tetra-decanoylphorbol-13-acetate (TPA, < 0.1% v/v) (Sigma-Aldrich) and incubated at 37 ± 1 °C in a humidified atmosphere of 5 ± 1% v/v CO_2_ in air for 24 h.

### BioFlux THP-1 cell adhesion assay

At the end of the incubation period, excess treatment solution was removed from all inlet and outlet wells. A sufficient volume of activated THP-1 cells at 5 × 10^6^ cells/mL was added to inlet well B and Hank’s Buffered Saline Solution (HBSS) (Gibco) was added to inlet well A. The plate was loaded onto the microscope stage with the BioFlux temperature controller set at 37 °C. THP-1 cells were perfused over the HAEC monolayer at 2 dyn/cm^2^ for 30 s followed by 0.5 dyn/cm^2^ for 1 min. Cells were incubated at 37 °C under gravity flow conditions for set adhesion periods and then washed with HBSS containing 10 µg/mL Hoechst 33342 (H33342) (Sigma-Aldrich) at 2 dyn/cm^2^ for 1 min, 5 dyn/cm^2^ for 1 min and 6 dyn/cm^2^ for 1 min. THP-1 cell perfusion, adhesion period and wash cycle was performed four times with increasing adhesion period length (10, 20, 30 and 40 min). Images were acquired using BioFlux Montage software (https://bioflux.fluxionbio.com/montage-software). Phase contrast images were captured from 3 fields of view per channel pre and post each wash step. After the final wash step, images were captured using the DAPI filter (420–480 nm).

Following the THP-1 adhesion assay cells were fixed in 3.7% paraformaldehyde (Sigma-Aldrich) in HBSS applied through inlet well B at 5 dyn/cm^2^ for 10 min at 37 °C. Excess paraformaldehyde was removed by washing in HBSS through inlet well A at 5 dyn/cm^2^ for 5 min. Cells were permeabilised in 0.1% Triton-X (Sigma-Aldrich) applied through inlet well B at 5 dyn/cm^2^ for 5 min and washed in PBS applied through inlet well A at 5 dyn/cm^2^ for 5 min. Non-specific binding was blocked by applying 5% BSA through the inlet wells at 2 dyn/cm^2^ for at least 60 min followed by washing in PBS at 5 dyn/cm^2^ for 5 min. Primary antibodies [ICAM-1 1:20 (R&D Systems, Abingdon, UK), CD11b 1:200 (Novus Biologics, Abingdon, UK)] in 1% BSA were applied through the outlet well at 2 dyn/cm^2^ for 2 min and then incubated overnight at room temperature. Appropriate secondary antibodies (anti-rabbit IgG (H+L) F(ab′)2 fragment Alexa Fluor 555 conjugate and anti-mouse IgG (H+L) F(ab′)2 fragment Alexa Fluor 488 conjugate (Life Technologies)) were applied through the outlet well at 2 dyn/cm^2^ for 2 min and then incubated at room temperature for 2–3 h. Cells were washed in PBS at 5 dyn/cm^2^ for 5 min and imaged at 40× magnification using a Zeiss LSM780 confocal microscope and Zeiss Zen software (https://www.zeiss.com/microscopy/int/products/microscope-software/zen.html). Co-localisation was assessed using ImageJ (https://imagej.nih.gov/ij/).

### Immunofluorescence microscopy

HAECs were seeded into 8-well Ibidi µ-slide chambered coverslips (Thistle Scientific; Glasgow, UK), pre-coated with 0.1% gelatin from porcine skin (Sigma-Aldrich) for 20 min at 37 °C. HAECs were seeded at 1 × 10^5^/mL in VECM and incubated overnight at 37 ± 1 °C, in a humidified atmosphere of 5% (v/v) CO_2_ in air. Confluent HAEC monolayers were treated with vehicle control (VECM), positive control (1 and 10 ng/mL TNFα) or eVCM, WSCM or nicotine [0, 15, 150, 1500 ng/mL nicotine equivalence (n.e.)] for 24 h at 37 °C. Six experiments with separate generations of WSCM, eVCM or fresh preparations of nicotine were performed. Cells were fixed in 3.7% paraformaldehyde for 10 min at 37 °C. Excess paraformaldehyde was removed by washing 3 times in PBS. Cells were permeabilised in 0.1% Triton-X for 4 min at room temperature and washed three times in PBS. Non-specific binding was blocked by incubating cells with 5% BSA for at least 60 min at room temperature followed by three PBS washes. Primary antibodies [ICAM-1 1:100, E-selectin 1:100 (R&D Systems)] in 1% BSA were applied to the cells and incubated overnight at room temperature. Secondary antibodies, anti-mouse IgG (H + L) Alexa Fluor 488 conjugate (Life Technologies) and DNA stain, H33342 were applied to the cells and incubated at room temperature for 2–3 h. Cells were washed three times in PBS and images captured with a Zeiss Axio Observer.Z1 microscope connected to a Hamamatsu CCD camera. Images were acquired using BioFlux Montage software. Three random fields of view were captured per well. Images were quantified using ImageJ to calculate fluorescence intensity of each image and divided by the number of cells per image to give average fluorescence per cell. Relative fluorescence was calculated by dividing average fluorescence at each treatment concentration by the vehicle control.

### Western blot

HAEC monolayers were grown in 96-well plates for 24 h prior to treatment with WSCM, eVCM or nicotine for an additional 24 h. Post-exposure cells were trypsinised and lysed in RIPA buffer. Five to ten micrograms (5–10 µg) of protein, as determined by Pierce BCA Protein Assay Kit (Thermo Fisher Scientific, Altrincham, UK), was loaded onto 4–12% Tris-Bis Gels (Invitrogen) and subjected to electrophoresis at 200 V (120 mA) for 1 h^8^. Separated proteins were transferred onto Amersham Protran Nitrocellulose membrane (GE Healthcare, Hatfield, UK) at 30 V (125 mA) for 1.5 h. Membranes were placed in Ponceau S solution (Sigma-Aldrich) to visualise protein bands and then washed in 1 × Tris Buffered Saline with Tween 20 (TBS-T; Cell Signaling Technology, Beverley, MA, USA) for 5 min on an orbital shaker^8^. Membranes were blocked with 5% Blotting-Grade Blocker (BioRad, Kidlington, UK) in 1 × TBS-T for 30 min^8^. Membranes were stored in 1% BSA TBS-T overnight at 2–8 °C. Membranes were incubated with primary antibodies rabbit anti-ICAM-1 1:1000 (Cell Signalling Technology, Danvers, MA, USA), rabbit anti-E-selectin 1:1000 (Novus Biologics), rabbit anti-VCAM-1 1:1000 (Cell Signaling Technology) or mouse anti-actin 1:5000 (Cell Signaling Technology) in TBS-T with 1% BSA for 4 h at room temperature, washed three times with TBS-T and incubated with horseradish peroxidase (HRP)-conjugated secondary antibodies donkey anti-rabbit 1:2500 or donkey anti-mouse (Stratech, Newmarket, UK) 1:10,000 in TBS-T for 1 h at room temperature. Membranes were washed three times in TBS-T at room temperature. Membrane surfaces were covered with a 1:1 mixture of freshly prepared ECL (SignalFire ECL; Cell Signaling Technology)^8^. After 1-min incubation, membranes were placed in a LI-COR C-DiGit Blot Scanner for chemiluminescence detection. Protein levels were quantified using Image Studio 5.x. Graphs represent ICAM-1 or E-selectin quantification relative to β-actin levels.

### siRNA transfection

HAECs were reverse transfected using lipofectamine RNAiMax (Invitrogen, Renfrew, UK,) in 75 cm^2^ flasks with siRNA duplexes. All ON-TARGET SMARTpool siRNA duplexes were used according to the manufacturer’s instructions (Dharmacon, Horizon Discovery, Waterbeach, UK). 270 μL of 2 μM siRNA was added to each flask. RNAiMax (72 μL) was incubated with 5.418 mL serum/antibiotic-free Gibco OptiMEM (Invitrogen) for 5 min at room temperature. The transfection reagent mix was added to the siRNA and incubated for 20 min at room temperature. Endothelial cells were seeded at approximately 1.5 × 10^6^ cells per flask in 12.48 mL serum/antibiotic-free OptiMEM. Cells were incubated for 6 h with siRNA duplexes at 37 ± 1 °C, in a humidified atmosphere of 5% (v/v) CO_2_ in air before replacing the media with fresh VECM. After 24 h, cells were seeded into a 24-well BioFlux plate and incubated at 37 °C for a further 24 h prior to test article treatment. After 24 h treatment, the BioFlux THP-1 cell adhesion assay was performed as described above and monocyte adhesion quantified. On the day of seeding into a 24-well BioFlux plate the remaining HAECs from each transfection were re-seeded into a 25 cm^2^ flask for cell lysis and protein analysis on the same day as the THP-1 cell adhesion assay was performed (72 h post-transfection).

### Statistical analysis

Statistical analysis was performed with GraphPad Prism. Comparisons between vehicle control and each concentration of WSCM, eVCM, nicotine or TNFα were performed using a one-way or two-way ANOVA with Dunnett’s multiple comparisons. Significant differences are shown on the relevant graphs as *p < 0.05, **p < 0.01, ***p < 0.001, ****p < 0.0001.

## Supplementary Information

Supplementary Information
