# Supplementary material for: Impact of cigarette versus electronic cigarette aerosol conditioned media on aortic endothelial cells in a microfluidic cardiovascular model
Source: Sci Rep. 2021 Feb 26;11:4747. doi: 10.1038/s41598-021-83511-7 (PMC7910588; doi:10.1038/s41598-021-83511-7)
Supplement: Supplementary file 1 — Supplementary Information [file 41598_2021_83511_MOESM1_ESM.pdf]

Impact of Cigarette Versus Electronic Cigarette Aerosol Conditioned Media on Aortic Endothelial Cells in a Microfluidic Cardiovascular Model

Om Makwana[3], Gina A Smith[1*], Hannah E Flockton[1], Gary P Watters[1], Frazer Lowe[2] and Damien Breheny[2]

[1]Covance Laboratories Ltd, Genetic and Molecular Toxicology, Harrogate, UK; [2]BAT R&D Centre, Southampton, UK, [3]Department of Internal Medicine, Section on Molecular Medicine, Wake Forest School of Medicine, Winston-Salem, NC 27157

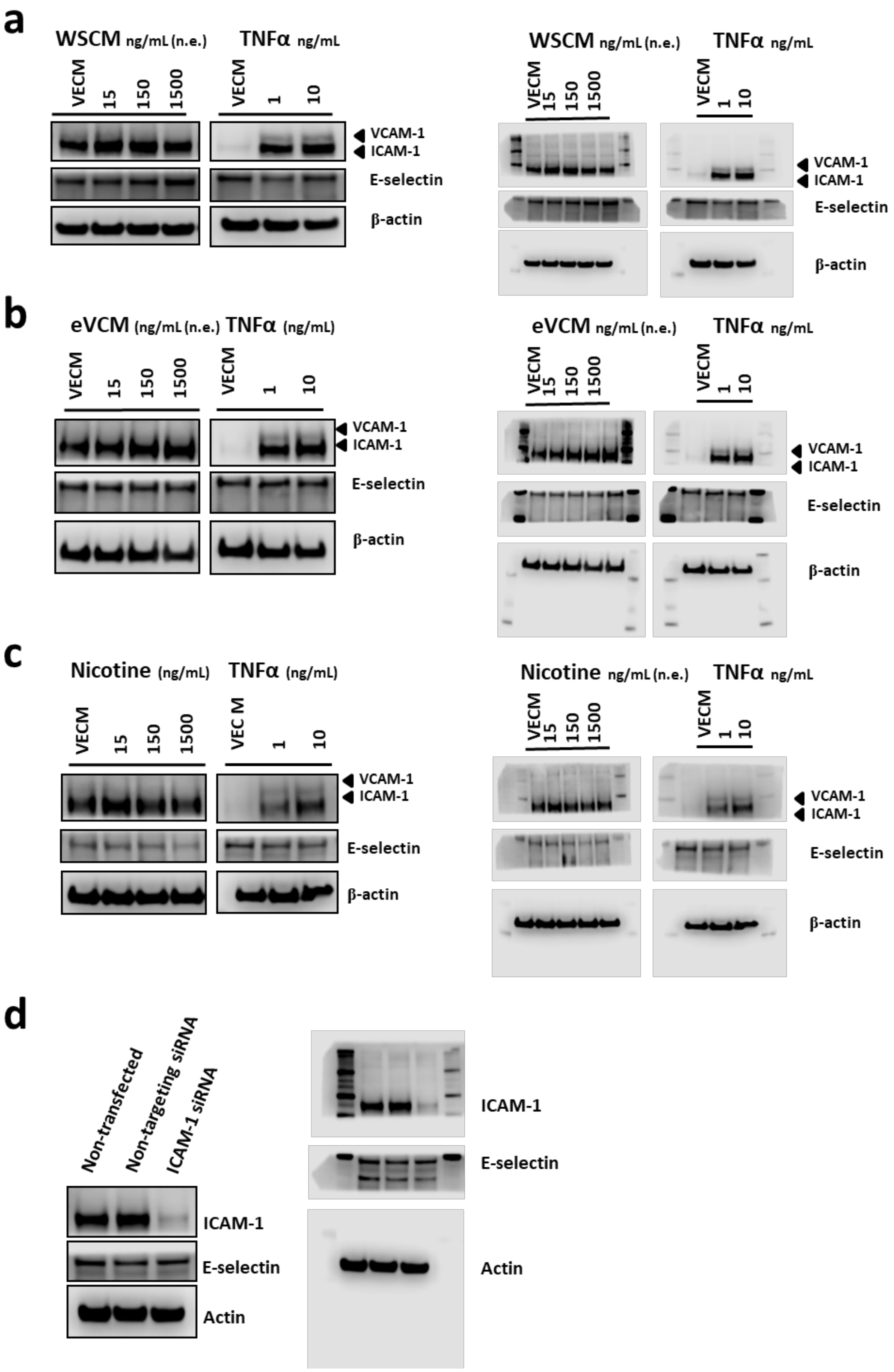

Supplementary Figure S1. Western blot images from A) Figure 3C, B) Figure 4C, C) Figure 5C and D) Figure 7C shown next to respective uncropped images.
